# Adherence to Treatment in Severe Asthma: *Predicting Factors in a Program for Asthma Control in Brazil*

**DOI:** 10.1097/WOX.0b013e3181d25e8e

**Published:** 2010-03-15

**Authors:** Adelmir Souza-Machado, Pablo Moura Santos, Álvaro A Cruz

**Affiliations:** 1 The Instituto de Ciências da Saúde, ProAR, Hospital Universitário Professor Edgar Santos, and ProAR, Faculdade de Medicina da Bahia, Universidade Federal da Bahia, Bahia, Brazil

**Keywords:** adherence, asthma, predictors, severe, treatment

## Abstract

**Background:**

In Brazil, like many other low- to middle-income countries, most asthmatic patients cannot afford the medication necessary to prevent exacerbations. The reference clinic of the Programme for Asthma Control in Bahia (ProAR; Salvador-Bahia) offers free medical care, pharmaceutical assistance (inhaled medication) and patient education. The reference clinic is accessible to all the population of Salvador and the Programme is targeted on severe asthma.

**Objective:**

The aim of the present study was to evaluate adherence to inhaled medication in severe asthmatics enrolled in ProAR.

**Methods:**

A sub-group of 160 consecutive severe asthmatics enrolled in ProAR were followed prospectively for 6 months. All patients were assessed by means the Asthma Control Questionnaire, the Beck Depression Inventory and spirometry. The rate of adherence to inhaled corticosteroid was checked monthly. A cut-off point of 80% of the doses prescribed in the period was used to define patients as adherent.

**Results:**

Of the one hundred-sixty patients with severe asthma included, it was possible to objectively assess adherence to the use of the inhaled corticosteroid in 158. Among these, 112 (70.9%) were considered adherent according to the adopted cut-off point. The rate of adherence in the whole sample of subjects was 83.9% of the prescribed doses. There was a significant association between asthma control and adherence to treatment. Predictors of poor adherence were adverse effects, living far from the referral center, limited resources to pay for transportation and dose schedule.

**Conclusion:**

In the present study, adherence to treatment was high. In a sample of patients with severe asthma managed in a public program that provides free medication and multidisciplinary treatment at a referral center, adherence to treatment was found to be associated with favorable clinical outcomes.

## 

Asthma is a chronic inflammatory disease characterized by symptoms such as dyspnea, chest tightness, wheezing, and cough. The goals of asthma treatment are (1) control of the clinical manifestations and (2) improvement in lung function [1].

There is evidence that reducing inflammation with therapy leads to clinical control. Inhaled corticosteroids are the most effective controller medications currently available [1]. However, asthma management and symptoms' control are not so easy to obtain. According to the World Health Organization, the definition of adherence is "the extent to which person's behavior--taking medication, after a diet and executing lifestyle changes, corresponds with agreed recommendations from a health care provider" [2].

Adherence to treatment is one of the most important factors that guarantees the success of the asthma treatment. Several factors may influence adherence to treatment and asthma control such as patients' knowledge about their disease, cultural and socioeconomic aspects, poor perception of asthma symptoms, adverse events, and skills in use inhaler devices [3-7]. In addition, compliance with medical prescriptions tends to decline steeply over time [8]. Patients with chronic diseases in high-income countries frequently do not use their medications as recommended by clinicians. In low- and middle-income countries, nonadherence is more critical because of the mix of limited access to health care, lack of appropriate diagnosis, and low availability or affordability of essential medicines [8].

The aim of this study was to review adherence and nonadherence to treatment patients with asthma, with emphasis on our experience in a reference center for control of severe asthma in Brazil.

## Methods

### Reference center of the programme for control of asthma in bahia (ProAR)

The reference clinic of the Programme for Asthma Control in Bahia (ProAR; Salvador-Bahia) offers free medical care, pharmaceutical assistance (inhaled medication), and patient education. The reference clinic is accessible to all the population of Salvador and the program is targeted for severe asthma. The patients use combined inhaled corticosteroid (beclomethasone or budesonide) and a long-acting *β*2 agonist (formoterol) for maintenance and also a short-acting inhaled *β*2 agonist (fenoterol) for rescue [1]. Additionally, patients and their families receive educational classes and multidisciplinary care by members of our team: pharmacists, nurses, psychologist, and clinicians.

A preliminary study comparing patients with severe asthma 1 year before and 1 year after admission to ProAR demonstrated that such intervention markedly reduced asthma morbidity in above 80% of these individuals [9]. (Table 1). This result suggests that regular use of prescribed medication was achieved.

**Table 1:** Utilization of Health Resources 1 Year Before and 1 Year After Admission in a Reference Centre for Asthma Control (ProAR) in Salvador-Bahia, Brazil: Analysis of a Subsample of 269 Consecutive Patients Completing 1 Year of Follow-up

	1 Year Before Admission	1 Year After Admission	*P*
Oral corticosteroid cycles	N = 1281	N = 421	< 0.01
	4.7 per patient	1.6 per patient	
Absenteeism with work (days)	N = 3056	N = 431	< 0.01
	11.4 per patient	1.6 per patient	
Visits to an emergency room	N = 8577	N = 1289	< 0.01
	31.9 per patient	4.8 per patient	
Hospitalizations	N = 383	N = 38	
	1.42 per patient	0.14 per patient	< 0.01

Reproduced with permission from *J Bras Pneumol *[9].

### Study design and patients' assessment

A subgroup of 160 consecutive patients with severe asthma enrolled in ProAR were followed prospectively for 6 months for objective quantification of their adherence to inhaled medication. Data about socioeconomic and demographic characteristics, history of asthma, severity of the symptoms based on the Asthma Control Questionnaire, [10] medications used, symptoms of depression (assessed by mean Beck Depression Inventory) [11] and beliefs, acceptance of the disease, knowledge of the benefits and risks of the treatment, and degree of communication with the health professionals who monitored the patient were collected. Patients also underwent pulmonary function tests (spirometry) for evaluation of the functional response to treatment.

At the end of each monthly visit, the patients received sufficient medication to use until the next visit, were instructed to fill a register with the missed doses, and return the packages of the medication dispensed in the previous visit. Powder capsules/inhalers returned were counted or weighed. The rate of adherence to the inhaled corticosteroid (IC) treatment was calculated as follows:

• For the ICs in capsule presentation, the following formula was used: (NU ÷ NT) × 100, where NU is the number of capsules actually used during the period and NT is the number of capsules that should have been used.

• For the ICs in dry powder presentation for oral inhalation, the inhalers where weighed at the time of dispensing and after 30 days of use, at which point the following formula was used: (U ÷ T) × 100, where U is quantity (in g) actually used during the period and T is the quantity (in g) that should have been used.

At the end of the follow-up period, the pharmacy database was analyzed to compare it with the information collected by the research assistants, and to determine the dates of patient visits and the quantity of medication dispensed at each visit. A cut-off point of 80% of the doses prescribed in the period was used to define patients as adherent. This value was adopted taking into consideration the severity of the disease and the availability of free medication to all of the patients treated via the ProAR.

### Ethical aspects

The study design and procedures were approved by the Ethics Committee for Research of the Federal University of Bahia School of Medicine. All patients gave written informed consent, and their identification data were maintained confidential.

### Statistics

Data were analyzed using the Statistical Package for the Social Sciences, version 9.0 (SPSS Inc., Chicago, IL, USA). The mean and SD were calculated for the quantitative variables that presented normal distribution.

Categorical variables were presented as frequency and proportion. The *χ*^2 ^test was used for the evaluation of the statistical significance among categorical variables and the Student's *t *test was used for the comparison among the means of the quantitative data. The Mann-Whitney *U *test and the Kruskal-Wallis test were used for the analysis of quantitative variables presenting asymmetric data. The logistic regression model was used for adjusting potential confounding factors.

The level of statistical significance was set at *α *< 0.05. The degree of association among the studied variables was evaluated using odds ratios with a confidence interval of 95%.

The calculation of the sample size was based on the following premises: total number of ProAR patients during the period (approximately 1600 patients); relative margin of error of 5%; confidence interval of 95%; prevalence of adherence of approximately 50%, based on data found in the literature; power of 80%; significance level of 5%; and a possibility of 20% loss to follow-up together, which indicated the ideal sample size to be 156 patients.

## Results

### General features of the sample followed up

Of the 160 patients with severe asthma included (Table 2), it was possible to objectively assess adherence to the use of the inhaled corticosteroid prescribed in 158 patients. Among these, 112 (70.9%) were considered adherent according to the adopted cutoff point. The rate of adherence in the entire sample of subjects was 83.9% of the prescribed doses.

**Table 2:** Socio-biologic Characteristics of the Sample of Severe Asthmatics From ProAR Studied Objectively for Adherence

Characteristic	Results (%)	Adherence (%)	*P*
Gender			
Male	40 (25%)	84.1	0.8
Female	120 (75%)	83.7	
Age (years), mean ± SD	49 ± 13.9		
No. children	2.7		
Religion, n (%)			
Catholic	94 (58.8%)	85.2	0.1
Protestant	34 (21.3%)	81.1	
Other	30 (19.9%)	83.8	
Permanent address	153 (95.6%)	83.8	0.4
Marital status, n (%)			
Married	75 (46.9%)	81.4	0.2
Single	46 (28.8%)	85.2	
Divorced	23 (14.4%)	86.4	
Widow/widower	16 (10%)	87.6	
Employment status, n (%)			
Employed	49 (30.6%)	78.4	0.7
Unemployed	31 (19.4%)	89.1	
Retired	37 (23.1%)	85.3	
Housewife	39 (24.4%)	84.3	
Level of education, n (%)			
Illiterate	15 (9.4%)	84.8	0.7
Junior high	89 (55.6%)	84.1	
High school	52 (32.5%)	83.1	
University	4 (2.5%)	83.9	

Reproduced with permission from *J Bras Pneumol *[7].

There was no difference in the mean age among the groups of adherent and nonadherent patients (49.7 and 46.8 years, respectively; *P *= 0.2). The mean duration of asthma in the studied sample was 26.0 ± 15.8 years, with a median of 26 years. The adherent patients had a longer asthma history in relation to nonadherent patients (27.8 and 22.1 years, respectively; *P *= 0.043; 95% CI: 0.18-11.19).

The rate of adherence of the patients residing in the city of Salvador compared with the patients residing outside Salvador was respectively 85.0% and 78.1%. (*P *= 0.01; 95% CI: 1.3-12.4).

Eighteen patients (11.3%) reported having limited resources to pay for transportation in the days scheduled for consultation. These patients presented lower adherence in relation to the other patients of this cohort (*P *= 0.013). The risk of nonadherence to treatment was 3.6 times higher among patients without resources for paying the fare for transportation (95% CI: 1.3-9.8).

### Asthma control

There was a significant association between asthma control and adherence to treatment. According to the Asthma Control Questionnaire (ACQ), 60 patients (38%) presented controlled asthma. ACQ scores were lower among adherent patients in comparison to nonadherent patients (1.6 versus 2.3; *P *= 0.008). Rate of adherence was higher among patients with controlled asthma in comparison to those with uncontrolled asthma (88.5% vs 80.9%; *P *< 0.008) The estimated risk of uncontrolled asthma was 2.9 times higher among nonadherent patients than among adherent patients (95% CI: 1.3-6.4).

### Predictors of poor adherence

Predictors of poor adherence were adverse effects, living far from the referral center, limited resources to pay for transportation, and dosing schedule (Table 3). Other factors of concern, such as symptoms of depression, religion, and economic status, were not associated with poor adherence in our study.

**Table 3:** Multivariate Analyses of the Variables Found as Predictors of Nonadherence in the Sample Studied in ProAR, Brazil

Variable	OR	Logistic Regression 95% IC	*P*
Residing outside Salvador	4.1	1.5-11.2	0.004
Longer duration of asthma	0.9	0.9-1.0	0.06
Occurrence of adverse event	3.9	1.3-11.5	0.01
Dose schedule: twice a day	5.8	2.2-14.8	0.0002
Transportation difficulties	4.0	1.3-12.8	0.017

Reproduced with permission from *J Bras Pneumol *[7].

### Visits to emergency room

Fifty-one of 158 patients (32%) reported emergency room visits in the course of the follow-up (mean ± SD = 3.2 ± 2.6 visits), and 18 (35%) of them were considered nonadherent to the treatment. One hundred seven patients (68%) did not visit the emergency room in the time period. Of those, 28 (26%) were considered nonadherent to the treatment. However, the overall adherence to treatment was not statistically different between groups who visited (81.5%) and did not visit (85.7%) the emergency room (*P *= 0.08) and the number of emergency room visits was not statistically different between adherent and nonadherent patients (0.9 ± 1.9 vs 1.4 ± 2.6 visits, respectively; *P *= 0.2).

### Scheduled doses of inhaled steroids

Inhaled corticosteroids were used 1 to 3 times a day: 123 (77.8%) patients used medication twice a day, 34 (21.5%) 3 times a day, and 1 (0.7%) only once a day. The mean daily dose of corticosteroids was 870 ± 200 *μ*g (median, 800 *μ*g), varying between 400 and 1600 *μ*g. Adherence to treatment was significantly lower among patients who used the IC 3 times a day than among those who used it 2 times a day (78.9% and 85.2%, respectively; *P *= 0.02; OR = 0.34; 95% CI: 0.22-0.68).

### Adverse events

Thirty-one (19.6%) patients reported adverse events after the use of inhaled medication. We observed an inverse association between the presence of adverse events and adherence to pharmacological treatment (*P *= 0.017). The estimated risk of nonadherence was 33% higher among patients who presented adverse events in the present study (95% CI: 0.1-0.7) in comparison to the patients who reported no adverse events. The adverse events most frequently reported by the patients were the following: epigastric pain in 3 patients; weight gain and heartburn in 2; and stomach acidity, agitation, conjunctivitis, oral candidiasis, pharyngeal irritation, nausea, throat clearing, hoarseness, drowsiness, tachycardia, dizziness, tremors, blurred vision, and xerostomia in 1.

### Depressive symptoms

Depressive symptoms were assessed by using Beck Depression Inventory (BDI). The mean ± SD for BDI score was 13.4 ± 10.0 (median, 11 points), and 54 individuals (33.8%) were classified as having depressive symptoms, scoring more than 20 points on the BDI. There was no significant association between adherence to treatment and depressive symptoms (*P *= 0.23). The rate of adherence was similar in both groups of patients (83%; *P *= 0.9).

### Other factors studied and not associated with adherence

In a logistic regression analysis adjusted for the variables that were considered statistically significant in the bivariate analysis, such as difficulty in communication with pharmacist and physician, access to medical assistance, religious and philosophical concerns, social embarrassment of using medication, and forgetfulness for using another medicine, we did not find statistically significant differences for any of the variables tested.

Only 14 patients (8.8%) reported having difficulty in understanding the information given by the physician and pharmacist who monitored them. The rate of adherence to treatment among those patients was 82.6%, and there was no significant difference between them and the other patients (*P *= 0.7). As for the access to medical assistance, 7 patients (4.4%) reported having difficulty in scheduling extra appointments when necessary. Among those patients, adherence to treatment was 85%, no different from the other participants (*P *= 0.8).

## Discussion

The present study showed the patients monitored in ProAR presented a high rate of adherence to asthma treatment (83.9%). Similar studies showed frequencies that vary from 30% to 70% [12]. In a national multicenter study that evaluated 131 people with asthma, Chatkin et al observed asthma therapy adherence in 51.9% of the patients [4].

One of the aspects of the present study that should be taken into consideration is that we observed good communication between patients and the health team who worked in ProAR, together with easy and rapid patient access to medical care and to extra appointments. In a study using different parameters to evaluate adherence to treatment, [13] patient satisfaction was the only factor significantly associated with adherence. In the present study, those aspects related to an empathic relationship between patients and health professionals contributed to compliance with pharmacological treatment.

Various factors can be considered predictors of nonadherence to treatment in patients with asthma. Poor knowledge of disease, lack of access to medication, difficulties in the understanding of proper medication use, and inhaler techniques contribute significantly to this problem, especially in low-income settings. In Lagos, Nigeria, specialists evaluated 106 patients with uncontrolled asthma and concluded that [14] the majority of the people with asthma did not receive any health education about their disease after consultation and 52.8% had poor inhaler technique. In the present study, we found an association between nonadherence to treatment and the following variables: adverse events; living far from the health facility; limited resources to pay the public transportation; and multiple daily doses. Asthma was better controlled in patients presenting higher adherence. In the present study, we were unable to evaluate the relationship between adherence to treatment and the different classifications of the severity of asthma because all patients selected had severe persistent asthma [1].

Depressive symptoms, as evaluated by the Beck Depression Inventory, presented no association with poor adherence to treatment. It is of note that the frequency of such symptoms was elevated among the asthma patients in the present study. The adopted cutoff point (20 points) for the evaluation of the depressive symptoms was more conservative than the one adopted in other studies in which the prevalence of depressive symptoms is high. This result differs from the findings of other studies in which depression has been associated with low adherence to treatment [15]. Corroborating our results, other studies showed a relatively high frequency of depressive symptoms in patients with more severe disease and presenting a low response to the treatment [16,17].

We used 3 different methods to assess adherence: pharmacy database records, counting or weighing medication, and diary registration. Therefore, overestimation of medication use was objectively evaluated.

Our study aimed to analyze adherence to treatment in a sample of patients with severe asthma who participated in a multiprofessional program of outpatient management, with free medication available. Consequently, the rates of adherence found in the present study cannot be extrapolated to the population in general because some predictors of adherence, such as the costs of the medication acquisition and of medical appointments, which can influence the pattern of their use, are not part of the scenario for the patients enrolled in this program. The use of inhaled corticosteroids contributes effectively and safely to the control of persistent asthma. Adherence to therapy is crucial to the success of the treatment. Studies of adults and children have shown that some 50% of those on long-term therapy fail to take medications as directed. Complete discontinuation of the asthma treatment will probably result in clinical deterioration and exacerbation and may contribute to poor clinical outcomes [13,18,19]. In the present study, the rate of nonadherence to treatment was higher among patients who had visited the emergency room. Although this difference did not rise to the level of statistical significance, there was a tendency toward a positive association between the number of visits to the emergency room and nonadherence, reflecting the inadequate asthma control among such patients.

The treatment regimen can influence the pattern of adherence. Our results confirm that patients that were prescribed a treatment regimen of 2 inhalations daily adhere more to treatment than those prescribed 3 daily inhalations. This result corroborates the findings of a systematic review of 76 clinical trials in which adherence was found to be inversely proportional to the frequency of daily doses [20].

Patient's apprehension about side effects of inhaled glucocorticosteroids may influence adherence in general and the number of doses effectively taken in a given day.

In a Brazilian survey, Chatkin et al observed adherence to asthma therapy in 51.9% of individuals. The authors also showed that the most severe asthma symptoms were associated with higher adherence to asthma therapy [4]. The reasons for patient's nonadherence are complex and are frequently related to patients themselves, health care professionals, the health care system, medications, and inhalation devices.

In the present study, the adverse events reported by the patients constituted another factor associated with low adherence to treatment. However, we did not observe corticosteroid phobia, which has been described in other studies, as a determining factor for adherence to treatment [21]. Although we found that a large number of patients feared the potential side effects of their medication, all patients stated that they considered the correct administration of the medication important. Furthermore, only half of these patients reported experiencing some adverse event which made it difficult to continue using the medication. Therefore, in our study, the patients failed to take their medication when they actually presented some adverse reaction to the medication.

The present study has some limitations in relation to its external validity, as mentioned before. We cannot extrapolate our results to the general population.

Another limitation of the study is the possible selection bias. By including the patients consecutively, we risk selecting only patients who came to the visits regularly, and it was therefore impossible to identify and interview the patients who missed visits. Nevertheless, during the study, adherence was evaluated prospectively and objectively, as were the many possible predictors of adherence, in patients with severe asthma.

Poor adherence is common and is closely associated with asthma morbidity. Nonadherence to treatment is not simply the result of a patients' deficiency. Better adherence results from overcoming obstacles such as access to medication, understanding of disease and its management, easy communication between health professionals and patients, and education programs fitted to health providers, patients, and their families.

## Conclusions

In a sample of patients with severe asthma managed by a public program that provides free medication and multidisciplinary treatment at a referral center, adherence to treatment was high and found to be associated with the clinical response to treatment. The most relevant predictors of poor adherence were living far from the clinic, limited resources for transportation, occurrence of adverse events, and daily multidose prescription.

## Note

Supported by FAPESB and CNPq, Brazil

ProAR, Centro de Saúde Carlos Gomes, Rua Carlos Gomes 270, 7o. andar 40060-330 Salvador, Bahia, Brazil
